# Antibodies against native proteins of *Mycobacterium tuberculosis* can detect pulmonary tuberculosis patients

**DOI:** 10.1038/s41598-023-39436-4

**Published:** 2023-08-04

**Authors:** Desak Nyoman Surya Suameitria Dewi, Ni Made Mertaniasih, Soedarsono, Kimika Hagino, Tomoya Yamazaki, Yuriko Ozeki, Wayan Tunas Artama, Haruka Kobayashi, Erina Inouchi, Yutaka Yoshida, Satoshi Ishikawa, Amina Kaboso Shaban, Yoshitaka Tateishi, Akihito Nishiyama, Manabu Ato, Sohkichi Matsumoto

**Affiliations:** 1 Department of Bacteriology, School of Medicine, Niigata University, Asahimachi-Dori 1-757, Chuo-ku, Niigata, 951-8510 Japan; 2 Department of Microbiology, Faculty of Medicine, Universitas Ciputra, CitraLand CBD Boulevard, Made, Kec. Sambikerep, Surabaya, 60219 Indonesia; 3 Department of Medical Microbiology, Faculty of Medicine, Universitas Airlangga, Jl. Mayjen Prof. Dr. Moestopo 47, Surabaya, 60131 Indonesia; 4 Laboratory of Tuberculosis, Institute of Tropical Disease, Universitas Airlangga, Kampus C Jl. Mulyorejo, Surabaya, 60115 Indonesia; 5 Sub-Pulmonology Department of Internal Medicine, Faculty of Medicine, Hang Tuah University, Komplek Barat RSAL Dr. Ramelan, Jl. Gadung No.1, Jagir, Surabaya, 60111 Indonesia; 6 Department of Biochemistry, Faculty of Veterinary Medicine, Universitas Gadjah Mada, Jl. Fauna 2 Karangmalang, Yogyakarta, 55281 Indonesia; 7 One Health/Eco-Health Resource Center, Universitas Gadjah Mada, Jl. Teknika Utara, Barek, Sleman, Yogyakarta, 55281 Indonesia; 8 Fukuyama Zoo, 276‑1, Fukuda, Ashida‑cho, Fukuyama, Hiroshima 720‑1264 Japan; 9 Department of Mycobacteriology, Leprosy Research Center, National Institute of Infectious Diseases, Aoba-cho 4-2-1, Higashimurayama-shi, Tokyo, 189-0002 Japan

**Keywords:** Bacteriology, Tuberculosis, Tuberculosis

## Abstract

Accurate point-of-care testing (POCT) is critical for managing tuberculosis (TB). However, current antibody-based diagnosis shows low specificity and sensitivity. To find proper antigen candidates for TB diagnosis by antibodies, we assessed IgGs responsiveness to *Mycobacterium tuberculosis* proteins in pulmonary TB (PTB) patients. We employed major secreted proteins, such as Rv1860, Ag85C, PstS1, Rv2878c, Ag85B, and Rv1926c that were directly purified from *M. tuberculosis*. In the first screening, we found that IgG levels were significantly elevated in PTB patients only against Rv1860, PstS1, and Ag85B among tested antigens. However, recombinant PstS1 and Ag85B from *Escherichia coli (E. coli)* couldn’t distinguish PTB patients and healthy controls (HC). Recombinant Rv1860 was not checked due to its little expression. Then, the 59 confirmed PTB patients from Soetomo General Academic Hospital, Surabaya, Indonesia, and 102 HC were tested to Rv1860 and Ag85B only due to the low yield of the PstS1 from *M. tuberculosis*. The ROC analysis using native Ag85B and Rv1860 showed an acceptable area under curve for diagnosis, which is 0.812 (95% CI 0.734–0.890, *p* < 0.0001) and 0.821 (95% CI 0.752–0.890, *p* < 0.0001). This study indicates that taking consideration of native protein structure is key in developing TB’s POCT by antibody-based diagnosis.

## Introduction

Tuberculosis (TB) is an infectious disease with high incidence, prevalence, and mortality. Globally, there were 10.6 million cases of TB in 2021. Moreover, in 2021, there were 1.6 million mortalities, including TB patients comorbid with HIV/AIDS. The mortality number among HIV-negative people was about 1.4 million, while that of HIV-positive people was 187,000^1–3^. In that same year, it’s also reported that the top 30 high TB burden countries are primarily developing countries which accounted for 87% of new TB cases globally^3^. Based on those data, TB still become a big health issue in the world. Accurate but cheap and easy point-of-care testing for TB is in demand in TB-endemic countries to control this deadly disease globally.

The sensitivity and specificity of previously established commercial diagnostic tools are questionable because of their inconsistent results in diagnosing TB. Moreover, recent TB diagnostic method has some limitations. The *M. tuberculosis* culture in Löwenstein-Jensen (LJ) or BACTEC™ MGIT™ 960 system is time-consuming while nucleic acid amplification tests (NAATs) like GeneXpert MTB/RIF are expensive. In addition, the WHO (2015) does not recommend the use of the tuberculin skin test (TST) and interferon-gamma release assay (IGRA) to diagnose active TB. Moreover, the IGRA and TST cannot predict an individual's risk of TB progression^4,5^. However, the immune response from pulmonary TB (PTB) patients has the potential to track TB disease progression.

Previous studies suggest that an asymptomatic person produces antibodies against *Mycobacterium tuberculosis (M. tuberculosis)* antigens in small amounts. In contrast, active tuberculosis patients exhibit increased antibody titers^5–7^. Thus, detection of patients’ immune response profile against *M. tuberculosis* antigens is a reasonable way to diagnose both active TB, and TB progression from latent tuberculosis infection (LTBI).

Bacterial infections can activate cellular immunity and secretion of antibodies by plasma cells to fight the infection. Previous data showed that PTB patients have increased serum immunoglobulin titers against mycobacterial antigens; only around 10% didn’t show any increase^8,9^. However, previously developed serological tests provided inconsistent results with highly variable sensitivity and specificity values. Accordingly, commercially available serodiagnosis tests have not been recommended to detect TB^3,4^.

Meanwhile, antibodies can be used to diagnose most infectious diseases. Furthermore, antibody response detection can be done quickly and easily at a low cost. This makes it potentially useful in high TB burden countries if it is sensitive and accurate. IGRA, which detects cellular responses, is cumbersome, time-consuming, and expensive, with limited use in high burden TB countries. In addition, it was illustrated that the proportion of people with a positive IGRA test who developed the disease during follow-up was less than 10%^3^. Thus, it is necessary to know why the detection of antibodies, which are supposed to be explicitly produced against pathogens, is not being used for accurate TB diagnosis.

It is known that secreted proteins are immuno-dominant since they directly interreact with immune cells without bacterial disruption. In this study, we assessed antibody responses against major secreted proteins purified from *M. tuberculosis*, such as Rv1860, Ag85C, PstS1, Rv2878c, Ag85B, and Rv1926c, in PTB patients from Soetomo General Academic Hospital, Surabaya, Indonesia. We chose to focus on these proteins because they are either characterized as major secretory proteins, highly immunogenic or are immunostimulatory components of the mycobacterial cell membrane. We found the importance of using native *M. tuberculosis* proteins in antibody-based TB diagnosis.

## Results

### Population characteristic

**Figure 1:**
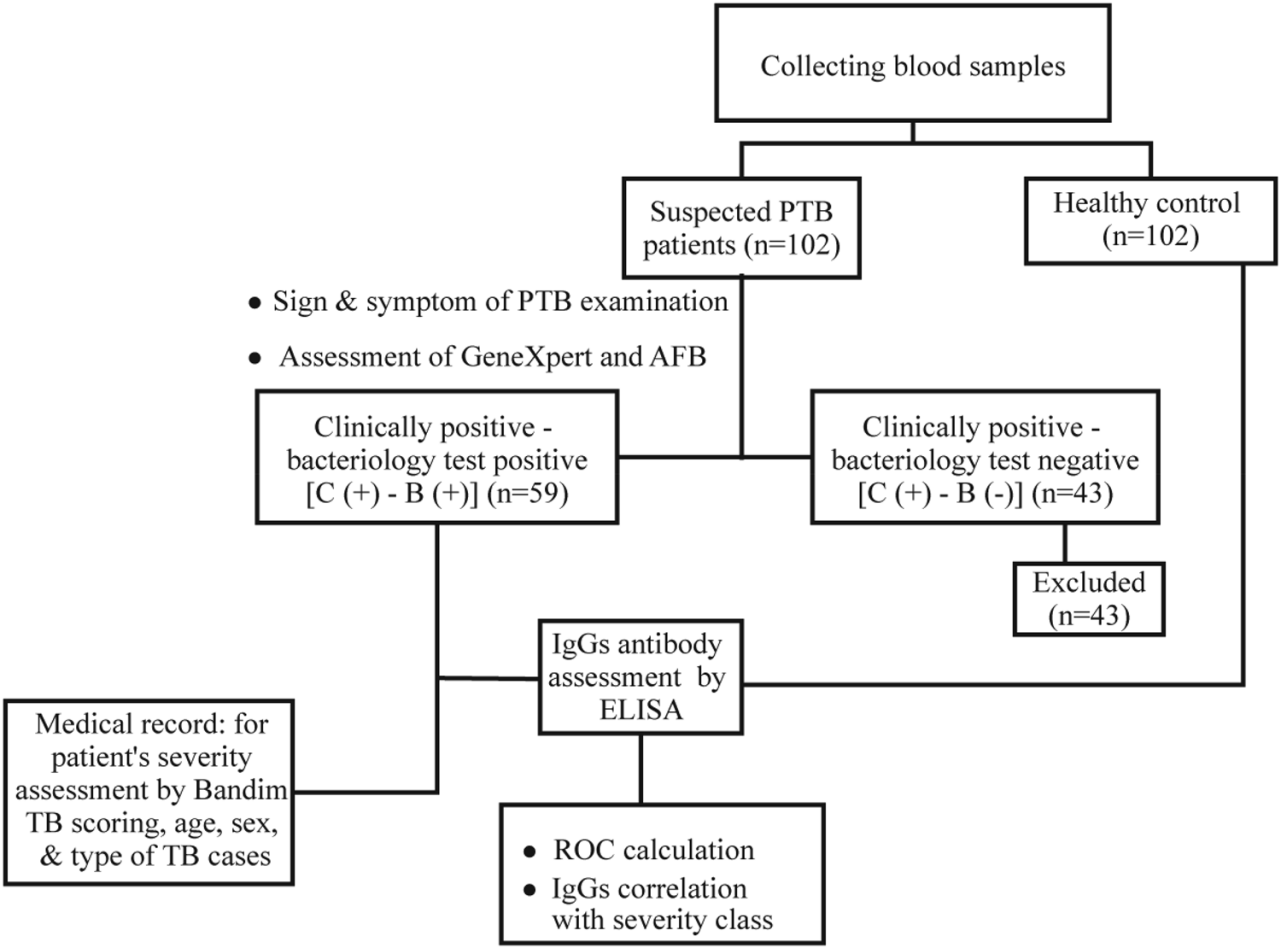
Flow diagram of study design and categorize of sample.

Fifty-nine serum samples from clinically positive—bacteriology test positive [C (+)–B (+)] group and 102 serum samples from the HC group were acquired (Fig. 1). Most patients in the C (+)–B (+) group were in the age range of 55–64 (29%). In this study, there were more female patients who had a higher percentage of TB infections than male patients. About 61% of patients from the C (+)–B (+) group were new cases. In addition, about 25% of patients in the C (+)–B (+) group had community-acquired pneumonia (CAP) as a comorbid (Table 1).

**Table 1:** Characteristics of PTB patients from Soetomo General Academic Hospital and HC.

Characteristic	C (+)–B (+) n = 59	HC n = 102
Age (mean ± SD)	46.41 ± 15.74	48.07 ± 15.97
Age range		
15–24	7 (12%)	10 (10%)
25–34	8 (14%)	12 (12%)
35–44	12 (20%)	22 (22%)
45–54	10 (17%)	19 (19%)
55–64	17 (29%)	22 (22%)
≥ 65	5 (8%)	17 (17%)
Sex		
Males	28 (47%)	44 (43.1%)
Females	31 (53%)	58 (56.9%)
Type of TB cases		
Relapse PTB	9 (15%)	
New cases PTB	36 (61%)	
MDR-TB	11 (19%)	
Loss to follow-up	2 (3%)	
Treatment failures	1 (2%)	
Comorbid PTB patients		
DM	9 (15%)	
CAP	15 (25%)	
Coronary artery disease	1 (2%)	
Pneumothorax	3 (5%)	
Bronchiectasis	1 (2%)	

*PTB* pulmonary tuberculosis, *MDR-TB* multidrug-resistant tuberculosis, *C (+)–B (+)* clinically positive—bacteriology test positive, *HC* healthy controls, *WHO* world health organization, *CAP* community-acquired pneumonia, *DM* diabetes mellitus.

### Screening for IgG in PTB patient sera that react to *M. tuberculosis* secreted proteins

**Figure 2:**
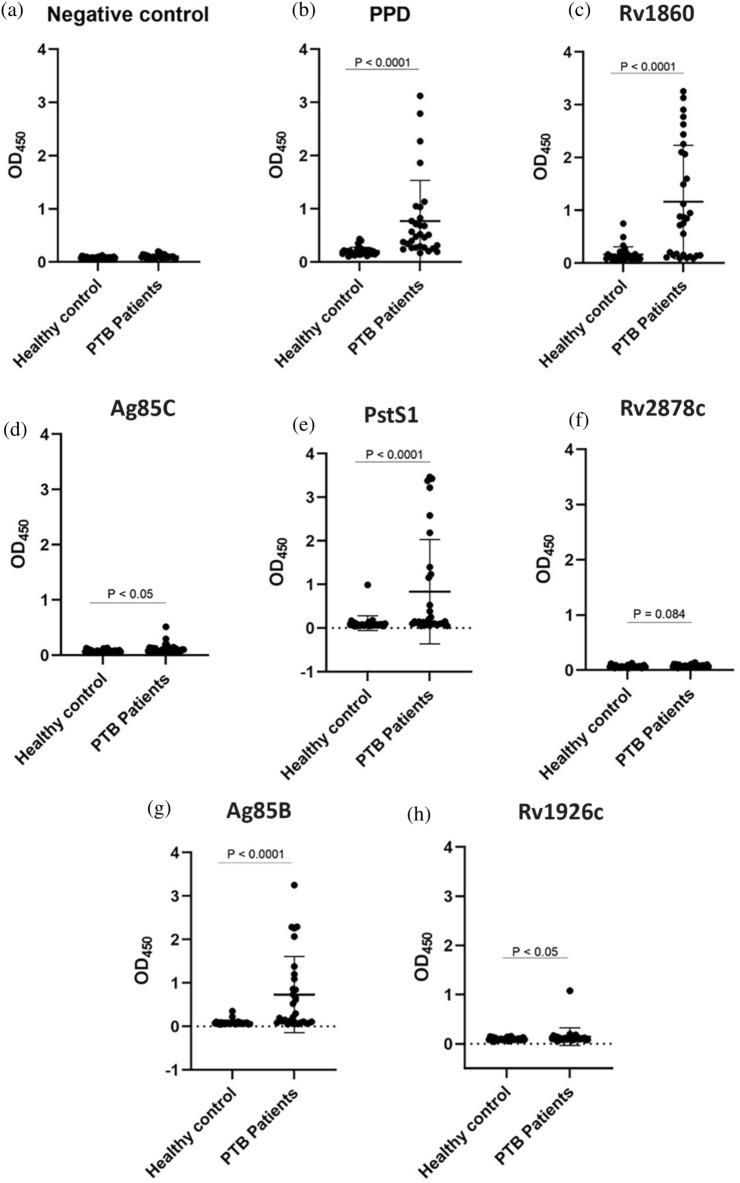
Graphic of the IgG amount in response to (**a**) negative control (no protein, containing PBS 1x), (**b**) PPD, (**c**) Rv1860, (**d**) Ag85C, (**e**) PstS1, (**f**) Rv2878c, (**g**) Ag85B, and (**h**) Rv1926c indicating the serum antibody concentration in 30 PTB patients and HC groups. The results were analyzed as individually and the data presented as mean ± SD.

We purified secreted proteins such as Rv1860, Ag85C, PstS1, Rv2878c, Ag85B, Rv1926c, and purified protein derivative (PPD) from *M. tuberculosis* culture filtrates, and tested their recognition by IgG from PTB sera using ELISA. We used PPD as a control since it represents the total heat-inactivated culture filtrates of *M. tuberculosis*. As a preliminary study, screening was done on sera from 30 PTB patients out of 59 PTB patients from Soetomo General Academic Hospital, and an equal number of HC out of 102 HC. The IgG antibody levels of the patients' group against PPD, Rv1860, PstS1, Ag85B, and Ag85C were significantly higher than those in the HC group. In contrast, IgG antibody titers against Rv2878c and Rv1926c antigens showed no significant differences between PTB patients and HCs (Fig. 2).

ROC analysis revealed that PPD, Rv1860, Ag85B, PstS1, and Ag85C had an acceptable AUC value among seven antigens (Supplementary Fig. S1). In contrast, Rv2878c and Rv1926c had a low AUC value (Table 2). The sensitivity of IgG antibody response to the seven antigens ranged from 53.3 to 83.33%. In addition, the positive predictive value (PPV) ranged from 69.57 to 95%, and the negative predictive value (NPV) ranged from 62.16 to 84.38%. Statistical results showed that PPD, PstS1, Ag85B, and Rv1860 have the potential to be used as antigen for serodiagnosis because they showed higher sensitivity, specificity, PPV, and NPV compared to the others. Although Ag85C has an acceptable AUC value, it has low sensitivity (Table 2). Taken together, these data suggest that IgGs recognize some, but not all proteins in active PTB patients.

**Table 2:** Individual ROC analyses, sensitivity, specificity, PPV, and NPV of seven antigens of *M. tuberculosis.*

Antigen	Concentration			ROC analysis			Sensitivity (%)	Specificity (%)	PPV (%)	NPV (%)
	PTB	HC	*p*-value*	AUC	*p*-value	Cut-off				
PPD	0.771 ± 0.764	0.205 ± 0.077	< 0.0001	0.914	< 0.0001	> 0.2665	83.33	90	89.29	84.38
Rv1860	1.161 ± 1.075	0.162 ± 0.146	< 0.0001	0.838	< 0.0001	> 0.5240	63.33	96.67	95	72.50
Ag85C	0.118 ± 0.091	0.074 ± 0.023	0.004	0.715	0.004	> 0.09350	53.33	86.67	80	65
PstS1	0.837 ± 1.196	0.114 ± 0.169	< 0.0001	0.798	< 0.0001	> 0.09500	80	76.67	77.42	79.31
Rv2878c	0.077 ± 0.022	0.068 ± 0.021	0.084	0.630	0.084	> 0.07300	53.33	76.67	69.57	62.16
Ag85B	0.733 ± 0.876	0.090 ± 0.059	< 0.0001	0.831	< 0.0001	> 0.1040	73.33	86.67	84.62	76.47
Rv1926c	0.144 ± 0.179	0.096 ± 0.025	0.019	0.676	0.019	> 0.1005	63.33	73.33	70.37	66.67

*ROC* receiver operating characteristic, *AUC* area under the curve, *PTB* pulmonary tuberculosis, *HC* healthy control, *PPV* positive predictive value, *NPV* negative predictive value.

*p-value by Mann–Whitney test.

### Comparison of recombinant and native PstS1 and Ag85B antigens

**Figure 3:**
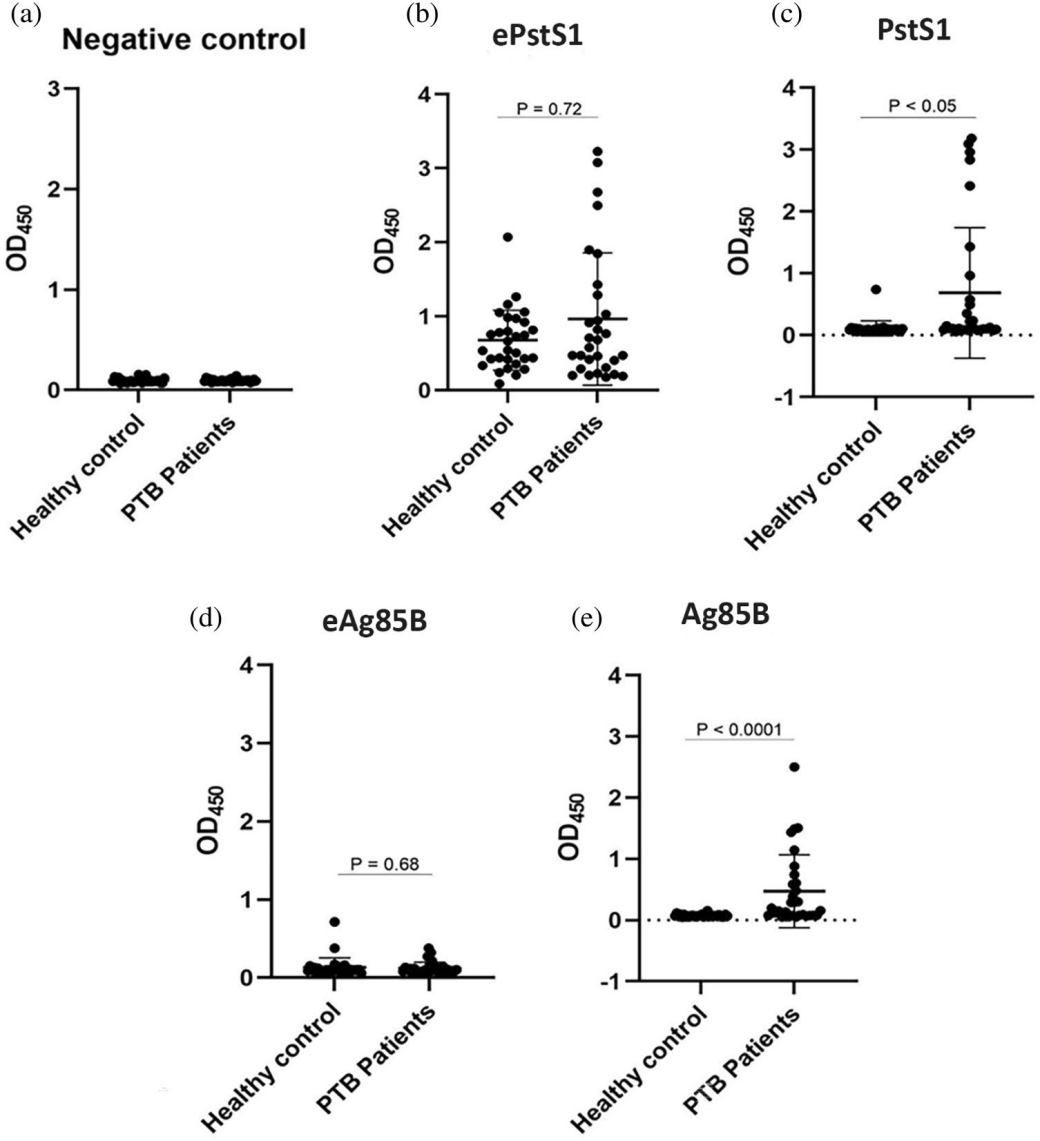
Graphic of the IgG amount in response to (**a**) negative control (PBS 1x), (**b**) ePstS1, (**c**) PstS1, (**d**) eAg85B, and (**e**) Ag85B antigen indicating the serum antibody concentration in 30 PTB patients and HC groups. The results were analyzed as individually and the data presented as mean ± SD.

The unexpected, highly sensitive and specific IgG levels to PstS1, Ag85B, and Rv1860 in PTB patients led us to check whether similar AUC values can be seen when using recombinant proteins produced by *Escherichia coli* (*E. coli*). We expressed and purified recombinant ePstS1 and eAg85B in *E. coli* ClearColi^®^ BL21. However, Rv1860 was not successfully expressed, possibly due to the lack of post-translational modification in *E. coli*^10^. Hence, its comparison against the native protein was not possible. Afterward, we evaluated IgG responses of the 30 PTB patients and HC, similar to the previous screening IgG experiment, against recombinant and native PstS1 and Ag85B antigens. The result showed that the IgG antibody titer against native PstS1 and Ag85B was significantly higher in PTB patients than HC. In contrast, recombinant PstS1 and Ag85B displayed no significant differences between the two groups. (Fig. 3).

**Figure 4:**
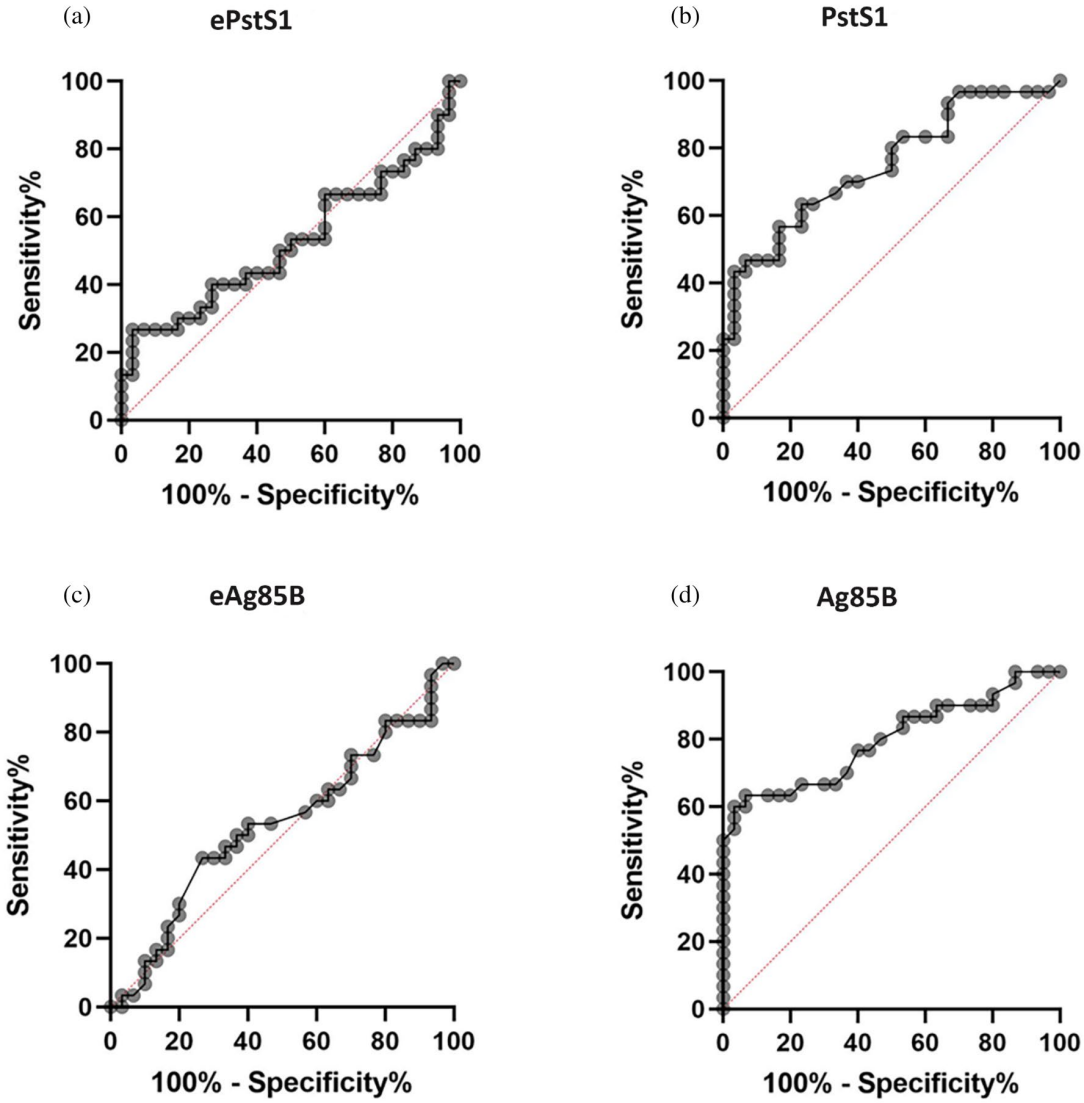
Receiver-operator characteristic (ROC) curve of IgG concentration of (**a**) ePstS1, (**b**) PstS1, (**c**) eAg85B, and (**d**) Ag85B antigen between PTB patients and healthy controls group.

We could not distinguish between PTB patients and HC by IgG titers when we used recombinant proteins expressed from *E. coli*. In the case of ePstS1, elevated IgG titers were observed in HC. In the case of Ag85B, eAg85B hardly reacted with IgG derived from PTB patient sera. Accordingly, ROC analysis shows that the AUC value of recombinant PstS1 and Ag85B was lower than native proteins, along with the sensitivity, specificity, PPV, and NPV (Table 3; Fig. 4). The result of this comparison indicated that the native protein performed better than the corresponding recombinant proteins.

**Table 3:** Individual ROC, sensitivity, specificity, PPV, and NPV of the native protein and recombinant protein of *M. tuberculosis.*

Antigen	Concentration			ROC analysis			Sensitivity (%)	Specificity (%)	PPV (%)	NPV (%)
	PTB	HC	*p*-value*	AUC	*p*-value	Cut-off				
ePstS1	0.963 ± 0.894	0.677 ± 0.405	0.727	0.527	0.723	> 1.277	26.67	96.67	88.89	56.86
PstS1	0.683 ± 1.055	0.109 ± 0.121	0.0007	0.749	0.0009	> 0.102	63.33	76.67	73.08	67.65
eAg85B	0.122 ± 0.078	0.131 ± 0.125	0.684	0.531	0.679	> 0.083	56.67	26.67	43.59	38.10
Ag85B	0.474 ± 0.595	0.077 ± 0.023	< 0.0001	0.797	< 0.0001	> 0.127	60	96.67	94.74	70.73

*ROC* receiver operating characteristic, *AUC* area under the curve, *PTB* pulmonary tuberculosis, *HC* healthy control, *PPV* positive predictive value, *NPV* negative predictive value.

*p-value by Mann–Whitney test.

### Profile of IgG responses between 59 PTB patients and 102 HC against three antigens

**Figure 5:**
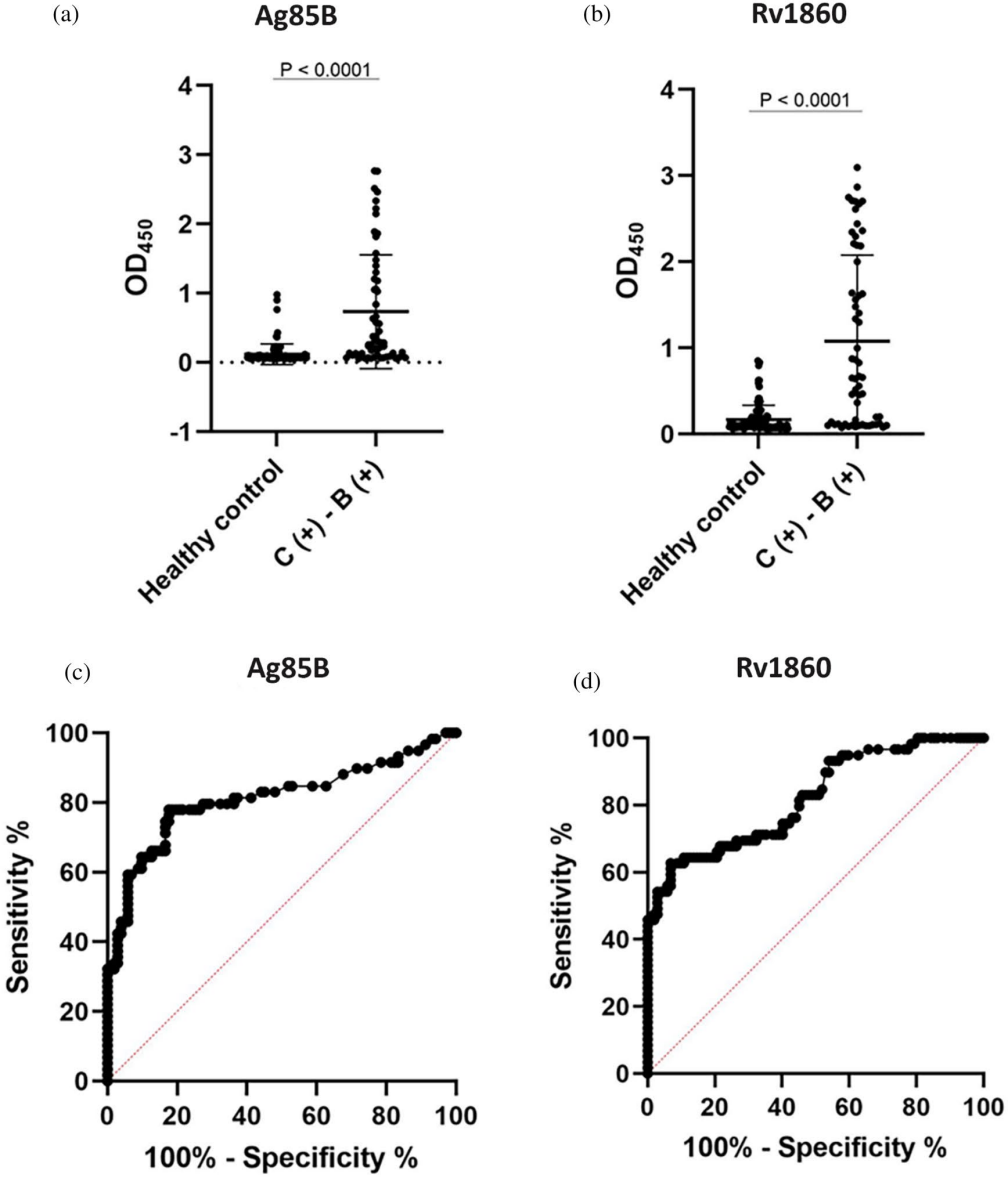
Graphic of the IgG amount in response to (**a**) Ag85B, (**b**) Rv1860, indicating the serum antibody concentration in the clinically positive—bacteriology test positive [C (+)–B (+)] and HC groups. The results were analyzed as individually and the data presented as mean ± SD. Receiver-operator characteristic (ROC) curve of the IgGs concentration of the (**c**) Ag85B and (**d**) Rv1860 antigen between clinically positive—bacteriology test positive [C (+)–B (+)] group and healthy controls showed that both antigens give acceptable value.

This study found that native Rv1860, Ag85B, and PstS1 reacted well with PTB patient IgGs in tested purified proteins. We continued further investigation by examining IgGs to native Ag85B and Rv1860 in higher number of subjects (59 PTB patients and 102 HC). We used native Rv1926c as a control for low immunogenic proteins. Unfortunately, we excluded PstS1 due to low yield of the native protein. We found that the concentration of IgG-class antibodies against Ag85B and Rv1860 in the C (+)–B (+) group was significantly higher than that in the HC group (*P* < 0.05, Fig. 5). However, antibodies against Rv1926c showed no difference between the HC and C (+)–B (+) (Table 4, Supplementary Fig. S4).

**Table 4:** Individual ROC analyses against the three antigens of *M. tuberculosis.*

Antigen	Concentration			ROC analysis		
	C (+)–B (+)	HC	*p*-value*	AUC	95% CI	*p*-value
Ag85B	0.735 ± 0.823	0.119 ± 0.149	< 0.0001	0.812	0.734–0.890	< 0.0001
Rv1860	1.077 ± 1.002	0.168 ± 0.166	< 0.0001	0.821	0.752–0.890	< 0.0001
Rv1926c	0.136 ± 0.167	0.100 ± 0.019	0.244	0.555	0.460–0.651	0.243

ROC, receiver operating characteristic; AUC, area under the curve; C (+)–B (+), clinically positive—bacteriology test positive; HC, healthy control.

ROC analysis between the C (+)–B (+) group and HC.

*p-value by Mann–Whitney test.

The ROC analysis from the three proteins showed that Ag85B and Rv1860 had AUC values of 0.812 (95% CI 0.734–0.890, *p* < 0.0001) and 0.821 (95% CI 0.752–0.890, *p* < 0.0001) (Table 4, Fig. 5) respectively, which are acceptable for diagnosis, while Rv1926c had a low AUC value (Table 4). Based on statistical analysis, Ag85B and Rv1860 showed higher sensitivity, specificity, PPV, and NPV than the other two antigens. The sensitivity value of Ag85B and Rv1860 was 77.97% and 62.71%, respectively. While the specificity of Ag85B and Rv1860 was around 82.35% and 93.41%, respectively (Table 5).

**Table 5:** Sensitivity, specificity, PPV, and NPV of antibodies against three antigens of *M. tuberculosis.*

No	Antigens	Cut-off	Sensitivity (%)	Specificity (%)	PPV (%)	NPV (%)
1	Ag85B	> 0.105	77.97	82.35	71.88	86.60
2	Rv1860	> 0.435	62.71	93.41	84.09	81.20
3	Rv1926c	> 0.108	38.98	79.41	52.27	69.23

Cut-off values were determined using Youden’s index. The highest value of Youden’s index was chosen as the best cut-off for the ROC curve.

*PPV* positive predictive value, *NPV* negative predictive value.

**Figure 6:**
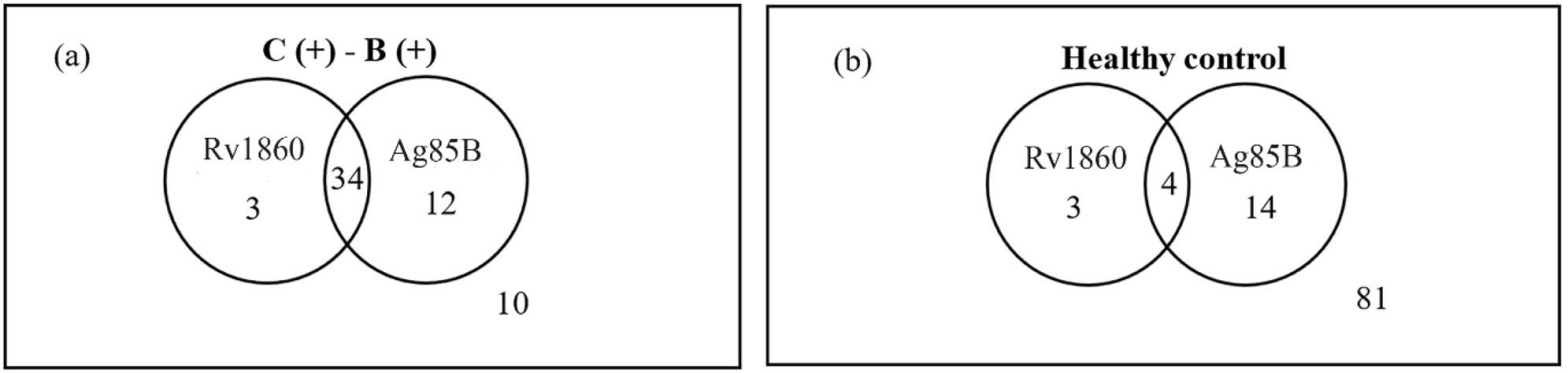
Venn diagram comparing Ag85B and Rv1860 data sets in (**a**) clinically positive—bacteriology test positive [C (+)–B (+)] and (**b**) healthy control (HC).

Based on the data, a Venn diagram was made to illustrate the relationship of IgG amount between Ag85B and Rv1860 against sera samples. The Venn diagram shows that in C (+)–B (+) group, about 34 patients were positive for both Ag85B and Rv1860 (Fig. 6) and in combination, resulted in sensitivity of 83%. On the other hand, 81 people from the HC group tested negative for both antigens, resulting in a combined specificity of 79.4%.

### No correlation between IgG responses and severity of PTB patients

We would like to show if the patients' disease severity affects their antibody response. Thus, we checked the correlation between the level of IgGs and the subject’s disease severity. The severity class (SC) of patients in this study was determined by Bandim TB scoring^11,12^. This scoring observed the signs and symptoms of patients to specify their severity class. Data showed that most patients had cough and dyspnea symptoms, while a few had a BMI of less than 16 kg/m^2^ (Table 6).

**Table 6:** Signs and symptoms of 59 research subjects from Soetomo General Academic Hospital.

Condition	59 research subjects
Symptoms	
Cough	52
Hemoptysis	11
Dyspnea	48
Chest pain	23
Night sweat	40
Signs	
Anemia	18
Pulse > 90 beats/menit	38
Positive of lung auscultation	37
Body temperature > 37 °C	38
BMI < 18 kg/m^2^	32
BMI < 16 kg/m^2^	8

*BMI* body mass index.

Based on the medical records, C (+)–B (+) patients were placed in the most severe group. Thirty-three C (+)–B (+) patients were classified in SC 3 group (Table 7). In addition, the correlation between severity class and IgGs response was analyzed by spearman.

**Table 7:** Percentage of C (+)–B (+) patients per severity class.

Severity class	C (+)–B (+) (n = 59)
SC 1	8 (13.6%)
SC 2	18 (30.5%)
SC 3	33 (55.9%)

*SC* severity class, *C (+)–B (+)* clinically positive—bacteriology test positive.

The spearman analysis results showed no correlation between disease severity and patient’s IgG response against Ag85B and Rv1860 (Table 8). Aside from these two antigens, statistical analysis also showed that the IgG response against Rv1926c does not correlate with the severity of the research subject (Table 9). Taken together, these results suggest that the level of IgG against a particular *M. tuberculosis* protein can be helpful in the detection of TB, but does not reflect a patient’s TB disease status.

**Table 8:** The correlation between severity class and patient’s IgG response to Ag85B and Rv1860.

Subject research	Severity class	IgG response					
		Cut-off Ag85B		*p*-value	Cut-off Rv1860		*p*-value
		< 0.105	> 0.105		< 0.435	> 0.435	
C (+)–B (+) (n = 59)	SC 1	0	8	0.399	1	7	0.449
	SC 2	5	13		8	10	
	SC 3	8	25		13	20	

Significant *p* value was less than 0.05 (*p* < 0.05).

*SC* severity class, *C (+)–B (+)* clinically positive—bacteriology test positive.

**Table 9:** The correlation between severity class and patient’s IgG response to Rv1926c.

Subject research	Severity class	IgG response		
		Cut-off Rv1926c		*p*-value
		< 0.108	> 0.108	
C (+)–B (+) (n = 59)	SC 1	6	2	0.232
	SC 2	12	6	
	SC 3	18	15	

Significant *p* value was less than 0.05 (*p* < 0.05).

SC, severity class; C (+)–B (+), clinically positive—bacteriology test positive.

## Discussion

Developing rapid and accurate TB diagnostics remains a challenge today. The gold standard for TB diagnosis, LJ-based culture, takes around four to eight weeks to get results^13^. Meanwhile, BACTEC™ MGIT™ 960 takes about 14 days, which prevents timely TB diagnosis making it a challenge in TB control^13^. Antibody-based diagnosis is widely used to detect infectious diseases^14–16^. It could be applied to TB diagnosis because of its rapidness and simplicity, especially in high TB burden and low-income countries, if the sensitivity and specificity level is of an acceptable value. Furthermore, collecting blood samples for antibody detection is easier than collecting sputum, as patients sometimes have a problem expectorating sputa^13–17^.

The development of diagnostics based on antibody detection in sera has been reported for many years, yet it still needs further research. The product of serodiagnosis in detecting active PTB is controversial because of varying result. However, some previous studies support the results of this research. Several past studies prove that serodiagnosis has the potential to track TB disease progression from asymptomatic infection^5,8,18–20^.

We evaluated IgG levels against purified secreted proteins from *M. tuberculosis* and confirmed that some proteins are well recognized by IgG produced by PTB patients (Figs. 2 and 3). Our results showed that Ag85B and Rv1860 had the highest AUC value among the tested antigens. The AUC value indicates the accuracy of a diagnostic test. The ROC curve is considered good when it has an AUC value of > 0.80^21–24^. Ag85B and Rv1860 also had the best sensitivity, specificity, NPV, and PPV values compared to other antigens, which highlights their potential use as diagnostic targets. The result of this study confirmed elevated production of antigen-specific antibodies in PTB patients.

Contrary to our findings, several studies reported that recombinant proteins gives a result as a biomarker for TB diagnosis ^5,18,24–27^. These prior studies showed that Ag85B and Rv1860 have potential use as biomarkers in the diagnosis of TB progression. Ag85B is highly conserved among other species of mycobacteria^28^. Past serological studies on immune-polymerase chain reaction (I-PCR) demonstrated that some proteins such as Ag85B, ESAT-6, and cord factor against PTB along with extrapulmonary tuberculosis showed positive results^29^. In addition, other proteins such as CFP-10, CFP-21, MPT-64, and PstS1 have been highlighted as potential targets for I-PCR and quantitative real time I-PCR (RT-I-PCR)^30,31^. Moreover, Rv1860 is a well-described secreted protein in *M. tuberculosis* and that influences the production of IFN-γ by CD4^+^ and CD8^+^ T cells^10,32^.

Unexpectedly, we found that native proteins were better recognized by IgGs compared to the recombinant ones produced in *E. coli,* as detected by ELISA (Fig. 4). These results suggest the possibility that post-translational modifications, specifically occurring in *M. tuberculosis,* determines the responsiveness of IgGs to native *M. tuberculosis* proteins. It is reported that Rv1860 is glycosylated on its threonine at position 27, and this post-translational modification was critical in recognizing the CD8-T cell clone established in an LTBI patient^10^. Similarly, O-glycosylation occurs on PstS1 (38 kDa antigen)^33^. These post-translationally modified proteins are known to be highly immunogenic. It is considered that immune responses that recognize species-specific modifications in pathogen's proteins more accurately detect pathogens than recognition of protein sequences alone and, therefore, may lead to adequate host responses against infections. As such, accurate recognition by host immune responses is also helpful for the development of diagnosis method.

Ag85B is also extensively studied as a highly immunogenic secretory protein in *M. tuberculosis,* but its post-translational modification has not been reported so far. Our preliminary data showed that denaturized Ag85B still possesses reactivity with IgGs in TB patients. This suggests that Ag85B-specific IgGs do not recognize three-dimensional structures, but recognize undefined post-translational modifications that occur on Ag85B. However, we cannot exclude the possibility that unnecessary obstructive post-translational modifications occur on expressed in *E. coli*. Although we need further research to determine the exact cause of this phenomenon, it is clear that taking into account the native structure of *M. tuberculosis* proteins is key in the development of antibody-based TB diagnosis.

Globally, Indonesia had the second-highest TB burden in 2021, with a high total incidence rate of 354 per 100,000 population^3^. It is estimated that a quarter of the world's human population has LTBI, the bulk of which comes from TB-endemic countries like Indonesia. It was thus not surprising that, 102 healthy subjects tested in this study included individuals with LTBI.

Antibody level is correlated with the amounts of antigens; therefore, it reflects disease progression and activity. In a recent study, we reported undetectable levels of IgG against Ag85B in an elephant diagnosed with LTBI. However, this levels sharply increased right before TB onset, after the long-term latency^34^. It has been proven that increasing antibody levels can potentially track TB disease progression in asymptomatic humans infection^5,18^. As such, we speculate that the observed increase in IgGs against Ag85B and Rv1860 among HC might indicate a TB development risk in LTBI populations.

LTBI is a significant source of TB. Precise antibody-based TB diagnosis can potentially detect not only active diseases, but also the risk of TB development from LTBI^5,18^. A longitudinal cohort study that investigates changes in IGRA and antibody levels among the LTBI population in Indonesia, is under consideration as a continuation of this study.

The measurement of severity level in PTB patients was done using the Bandim TB method. We tried to check the correlation between IgG level and PTB patient disease severity. According to Rudolf^12^, Bandim TB is a simple method used in middle—to low-level-income countries. The method helps monitor PTB or MDR-TB patients who are still in the medication period and can also have a role in screening TB disease^12^. That was our reason for using this method to determine the severity level in PTB patients. In this study, we did not include the mid-upper arm circumference (MUAC) measurement. The total point of this method was 13, if MUAC measurement was included, but turned 11 in this research^12^.

Our study evidenced that patient’s disease severity level doesn't influence their IgG response. A previous study also reported that scoring methods such as KPS, Bandim TB score 1, and Bandim TB scores II do not correlate with the cavitary disease on lung X-ray^35^. On the contrary, Niki et al.^18^ showed significant association of IgA levels against HrpA in active disease patients with clinical inflammation status measured by “C-reactive protein (CRP) at entry”, entry was meant as the point of diagnosis before treatment. However, still according to Niki et al.^18^, there is no association between the IgG titers with the immunology indicator and other clinical indicators that they checked, such as severity degree based on X-ray type (cavity) and X-ray extent.

The correlation between the severity degree determined by Bandim TB and IgG response has not yet been reported. Based on previous research, it has been shown that nutrition status is vital in clinical TB manifestation. High TB incidence and prevalence is seen more in mid and low-level income countries compared to high-income countries. Thus, seeking a relation between nutrition status and IgG titers could be compelling for future research^18^.

To date, WHO still discourages the use of serological tests for active TB diagnosis; however, they encourage conducting further research to improve their quality^4,26^. Thus, this research can hopefully provide helpful information about the importance of native protein usage for antibody-based TB diagnosis. Subsequent research about native *M. tuberculosis*-specific proteins, such as ESAT-6 and CFP-10, might be valuable.

Based on the presented result, we deduce that combined rather than single antigen testing might be a decent diagnostic strategy for developing a serodiagnosis assay that could also predict TB progression. In the future, we plan to compare the sensitivity and specificity between serodiagnosis and IGRA in active TB, latent tuberculosis infection (LTBI) groups, and healthy people. Moreover, the serological test is also expected to support the diagnosis of other types of hard-to-diagnose TB, such as smear-negative PTB cases and extra-pulmonary TB, including TB pleuritis and TB meningitis. Thus, research about serological tests in diagnosing these types of TB could be valuable to develop. In addition, further examination of the prediction of post-translational modification of the proteins in *M. tuberculosis* is also important to be completed.

## Conclusion

The present study revealed that native protein performed better than recombinant one. The examination results specified that the native protein of *M. tuberculosis* is essential as a diagnostic candidate for tuberculosis. The results also demonstrated that native Ag85B and Rv1860 can distinguish between C (+)–B (+) and HC groups better than other proteins.

## Materials and methods

### Study population

The population of this study includes 102 suspected TB patients with clinical symptoms from Soetomo General Academic Hospital, Surabaya, Indonesia. All of the patients were examined with TB diagnostic tools, and 59 patients who were positive for the GeneXpert MTB/RIF (Cepheid, USA) test^36,37^ or/and acid-fast bacilli (AFB) were retrospectively selected as clinically positive – bacteriology test positive [C (+)–B (+)]. While 43 samples showed negative GeneXpert MTB/RIF results, they showed positive radiological result and exhibit positive sign and symptoms of TB diseases(s). The 43 samples were termed clinically positive – bacteriology test negative [C (+)–B (−)] patients. Although these patients did not meet the criteria for bacteriological diagnosis, they all had abnormal results in the chest X-ray examination supporting TB positive results. The doctor diagnosed them as active TB patients and decided to give them TB treatment^38,39^. However, we eliminated the 43 samples and focused on 59 of the [C (+)–B (+)] group (Fig. 1) because we couldn't prove that they were TB positive.

A total of 102 serum samples from healthy people or healthy control (HC), not showing any TB symptoms, were also collected (Fig. 1). The age range of HC was matched to those of the suspected TB patients. Thus, this study is an age-match case control study. Chest X-ray was performed on healthy subjects to confirm that they didn't have TB disease.

### Ethical statement

All methods in this study were implemented under the applicable guidelines and regulations. Healthcare workers of Soetomo General Academic Hospital collected all sera used in this study following the Declaration of Helsinki. The ethics committee of Soetomo General Academic Hospital approved the study with an ethical clearance number 0410/KEPK/VII/2018. Informed consent was obtained from all subjects or their legal guardian(s).

### Protein preparation

PPD was purchased from the Japan BCG laboratory (Tokyo, Japan). In addition, we purified some native proteins from *M. tuberculosis* (Supplementary Fig. S2). These proteins were acquired by a previous study using purified broth culture of *M. tuberculosis*^40^. However, upon further examination, Thioredoxin and Rv3803c were of low concentration so we exclude them from subsequent experiments.

Genes coding for PstS1, Ag85B, and Rv1860 proteins were amplified by polymerase chain reactions (PCR) for cloning. For expression of Ag85B, PstS1, and Rv1860 with a HIS-tag at their C-terminal ends, we synthesized primers containing 6 × HIS sequence and restriction enzyme sites (Nde1 and EcoR1), and amplified targeted DNA regions from genomic *M. tuberculosis* DNA by PCR. The primers used included Ag85B-HIS forward: 5′-CC**CATATG**TTCAGCCGTCCGGGTCTGCCGG-3′; Ag85B-HIS reverse: 5′- CC**GAATTC**TATTAGTGGTGGTGGTGGTGATGACCCGCACCCAGGCTG-3′; PstS1-HIS forward: 5′-CCC**CATATG**GGTTGCGGCAGCAAGCC-3′; PstS1-HIS Reverse: 5′-CC**GAATTC**TATTAGTGGTGGTGGTGGTGGTGGCTGCTAATGGTCGCGATCAG-3′; and Rv1860-HIS forward: 5′-CC**CATATG**GACCCGGAGCCGGCTCCGCC-3′; Rv1860-HIS Reverse; 5′-CC**GAATTC**TATTAGTGGTGGTGGTGGTGGTGCGCCGGCAGGGTACGTTG-3′.

Amplified DNAs were digested with Nde1 and EcoR1 resulting in DNA fragments containing Ag85B, PstS1, or Rv1860 gene, which were purified by fractionation with gel electrophoresis followed by insertion into the same site in pET22b (+) (Novagen). After confirming the DNA sequence of each expression vector by the Sanger method, expression vectors were transformed into ClearColi^®^ BL21. Transformants were cultured in 500 mL Luria–Bertani (LB) media containing 50 µg/mL carbenicillin at 37 °C to an optical density of 0.5. For recombinant protein expression, isopropyl β-d-1-thiogalactopyranoside (IPTG) was then added to a final concentration of 0.5 mM and the culture further incubated for 1 h.

Bacterial cultures were immediately cooled in ice and collected by centrifugation at 8000 g for 10 min at 4 °C. After removing the supernatant; bacterial pellets were suspended in 10 mM Tris–HCl and 300 mM NaCl buffer pH 7.5 and disrupted by sonication with cooling. The samples were then centrifuged at 10,000 g for 10 min at 4 °C, and solubilized fractions and pellets were separated. At this point, we performed sodium dodecyl sulfate–polyacrylamide gel electrophoresis (SDS-PAGE) and that the majority of eAg85B and ePstS1 were found in the pellets. On the other hand, despite multiple attempts, we could not detect the expression of eRv1860 and hence could not proceed with its purification.

The pellets, including eAg85B and ePstS1, were solubilized in 10 mM Tris–HCl, 300 mM NaCl, and 6 M Urea and applied to Ni‐NTA column by His GraviTrap™ (GE Healthcare Amersham Bioscience, UK), and purified in the presence of 6 M Urea according to the manufacturer’s instruction. The urea concentration was then gradually lowered by dialysis from 6 to 0 M over the course of a week to facilitate protein refolding, and samples were finally suspended in a solution of 10 mM Tris–HCl and 300 mM NaCl. The purified ePstS1 and eAg85B were then analyzed by electrophoresis (Supplementary Fig. S3). The results were validated by mass spectrometry (Supplementary Fig. S3).

### Proteomic analysis

Proteomic analysis was done to further identify the purified native proteins from *M. tuberculosis* and recombinant protein (ePstS1and eAg85B) from *E. coli* (Supplementary Figs. S2 and S3). Purified preparation of the native proteins and recombinant protein was separated by electrophoresis on a polyacrylamide gel, and then stained with Coomassie Brilliant Blue R250. Each purified protein band was excised, reduced, and alkylated with dithiothreitol and iodoacetamide respectively, and subjected to in-gel trypsin digestion as described previously^41,42^.

The trypsin digests were dissolved in 0.3% formic acid, filtered through a 0.45 µM membrane filter (Ultrafree-MC, Millipore) and analyzed under direct injection mode on an Eksigent NanoLC 415 nano-flow liquid chromatography system (AB Sciex, Framingham, MA, USA) using a 75 µm × 150 mm C18-spray tip column (3 µm, 120 Å, Nikkyo Technos, Tokyo, Japan) coupled with a TripleTOF5600 + tandem mass spectrometer (AB Sciex).

Mobile phase A was 0.1% formic acid. While, mobile phase B was 0.1% formic acid in acetonitrile. Peptides were eluted by a 20 min gradient of 2% to 32% B at 300 nL/min. The MS spectrum and 10 MS/MS spectra were acquired in a data-dependent mode with 1.3 s of cyclic time. Dynamic exclusion time was set at 8 s. Auto-calibration was carried out every 4–5 samples using 50 fmol bovine serum albumin (BSA) trypsin digest as a calibrant (KYA technology, Tokyo, Japan).

The raw data produced by Analyst TF 1.6 software (AB Sciex) was converted to mascot generic files by MS Data Converter (AB Sciex). It was then searched against an in-house build *Mycobacterium tuberculosis* (strain ATCC25618/H37Rv) protein sequence database downloaded from UniProt on November 7, 2016, using a Mascot search engine (version 2.6, Matrix Science, Boston, MA, USA). Peptide and MS/MS tolerances were set at ± 20 ppm and ± 0.1 Da, respectively. A maximum of 2 missed cleavages was allowed. Modifications were put as follows: carbamidomethylation on cysteine was the fixed modification, while deamidation of asparagine or glutamine, oxidation of methionine, N-terminal glutamine to pyroglutamate, and N-terminal glutamic acid to pyroglutamate were included as variable modifications. The target false discovery rate (FDR) was set at < 1%.

### Enzyme-linked immunosorbent assay (ELISA)

ELISA was used to determine the IgGs concentration from PTB patients and HC against several proteins of *M. tuberculosis*. The antigen was diluted in phosphate buffer saline (PBS) 1 × pH 7.2 to the concentration of 5 μg/mL. The microplates (Maxisorp, Thermo Scientific Nunc, Denmark) were then coated with the antigens overnight at 4 °C. Afterwards, the plates were washed with PBS-Tween 20 and then blocked with 5% skimmed milk in PBS containing 0.05% Tween 20 overnight at 4 °C^43^.

The next step was to add human serum samples diluted 1:200 in PBS containing 0.05% Tween 20 and 1% skimmed milk into plates and incubated at 37 °C for 1 h. The plates were then incubated for 1 h with goat anti-human IgG Fc-HRP (Southern Biotech, Cat No 2048-05) at 1:5,000 dilution. Next, 100 μl SureBlue reserve-TMB (SeraCare, 5120-0083, USA) was added to each well. After incubation for 5 min in the dark, the reaction was stopped by adding 100 μl 1N HCl. The absorbance was read using an iMark™ Microplate Absorbance Reader (Bio-Rad) at 450 nm^43^.

### Bandim TB scoring

PTB subjects enrolled in this study were clinically assessed, and their disease severity was classified using the modified Bandim TB scoring. The assessment criteria were based on the disease clinical manifestation of five symptoms (cough, hemoptysis, dyspnea, chest pain, and night sweat) and five signs (anemia, pulse > 90 beats/min, positive of lung auscultation, body temperature > 37 °C, body mass index (BMI) < 18 or < 16 kg/m^2^). In this study, the MUAC < 220 or < 200 mm was not included because the data was unavailable in Soetomo General Academic Hospital^12,44^.

Each variable is worth one point; specifically, the BMI variable gets an additional one point if the BMI is less than 16 kg/m^2^, so the maximum score is 11. There are three severity classes based on Bandim TB scoring that were applied in this study, namely: mild class or severe class 1 (SC 1) with a score of 0–3, moderate class or severe class 2 (SC 2) with a score 4–5 and severe class 3 (SC 3) with score 6–11^11^.

### Statistical analyses

Statistical results were analyzed by Mann–Whitney test using GraphPad Prism ver. 9.5.1 (GraphPad Software, San Diego, CA, USA), and results with *p* < 0.05 were considered significant. The receiver operating characteristic (ROC) curve and the area under the curve (AUC) with 95% CI for each antigen were also calculated using the same software. Youden's index determined the sensitivity and specificity of each antigen^43^. The result of IgG response with severity class was correlated using Spearman by SPSS.

### Supplementary Information

Supplementary Figures.

## Data Availability

The datasets in this study are available on reasonable request to the corresponding author.
