# Supplementary material for: Antibodies against native proteins of Mycobacterium tuberculosis can detect pulmonary tuberculosis patients
Source: Sci Rep. 2023 Aug 4;13:12685. doi: 10.1038/s41598-023-39436-4 (PMC10403504; doi:10.1038/s41598-023-39436-4)
Supplement: Supplementary file 1 — Supplementary Figures. [file 41598_2023_39436_MOESM1_ESM.pdf]

Supplementary Fig. S1

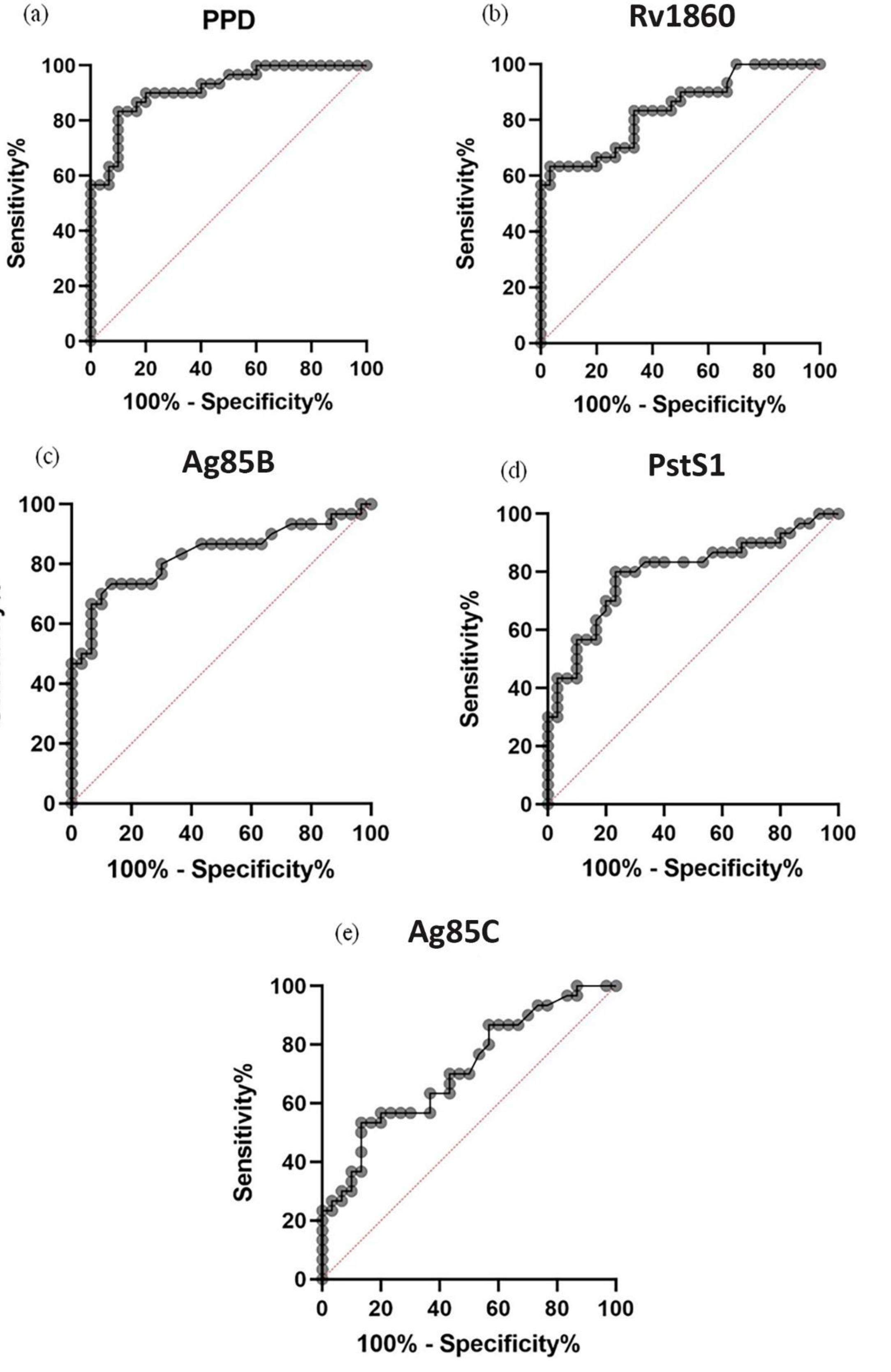

Receiver-operator characteristic (ROC) curve of the IgG concentration of the (a) PPD, (b) Rv1860, (c) Ag85B, (d) PstS1, and (e) Ag85C antigen between 30 TB patients and healthy controls.

Supplementary Fig. S2

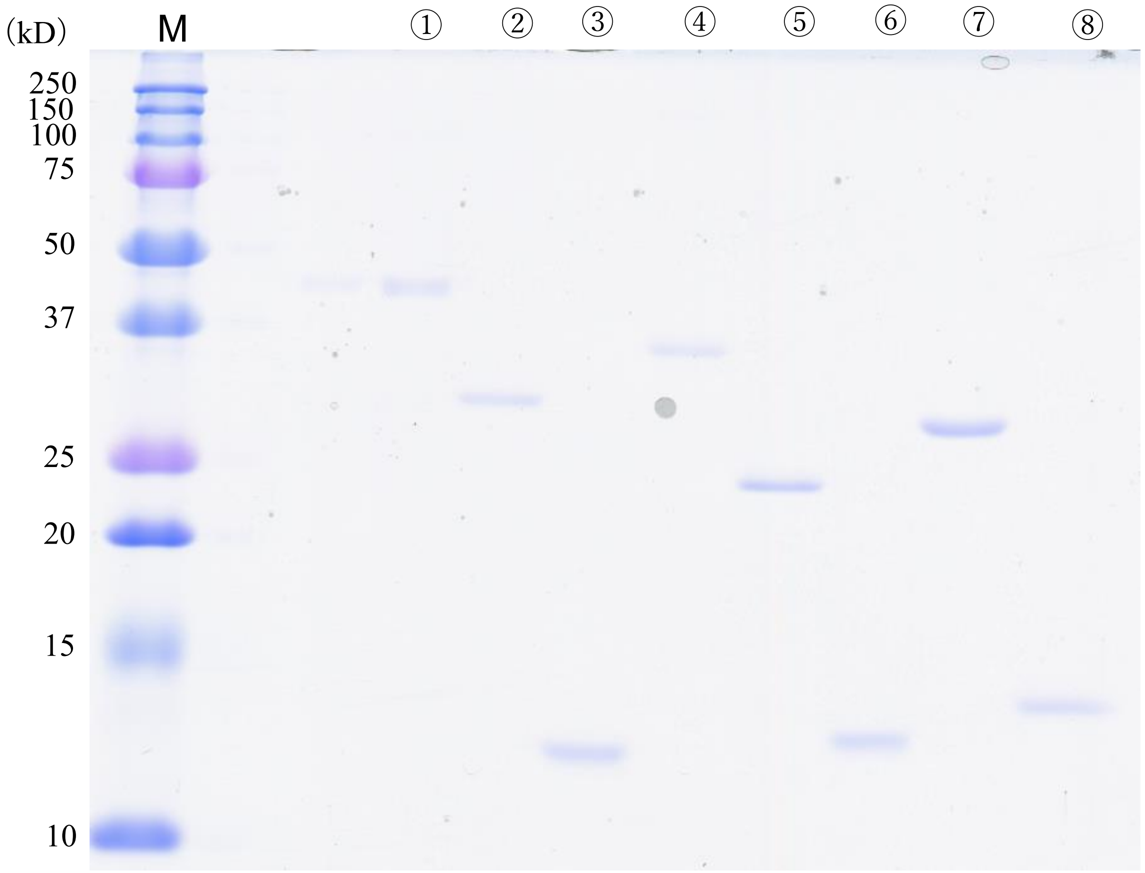

① Rv1860

Protein sequence coverage: 52%

Matched peptides shown in ***bold red***.

1 MHQVDPNLTR RKGRLAALAI AAMASASLVT VAVPATANAD PEPAPPVPTT

51 AASPPSTAAA PPAPATPVAP PPPAAANTPN AQPGDPNAAP PPADPNAPPP

101 PVIAPNAPQP VRIDNPVGGF SFALPAGWVE SDAAHFDYGS ALLSK**TTGDP**

151 **PFPGQPPPVA NDTRIVLGRL DQKLYASAEA TDSKAAARLG SDMGEFYMPY**

201 **PGTRINQETV SLDANGVSGS ASYYEVKFSD PSKPNGQIWT GVIGSPAANA**

251 **PDAGPPQRWF VVWLGTANNP VDK**GAAK**ALA ESIRPLVAPP PAPAPAPAEP**

301 **APAPAPAGEV APTPTTPTPQ** RTLPA

② Ag85C

Protein sequence coverage: 32%

Matched peptides shown in ***bold red***.

1 MTFFEQVRRL RSAATTLPRR LAIAAMGAVL VYGLVGTFGG PATAGAFSRP

51 GLPVEYLQVP SASMGRDIK**V QFQGGGPHAV YLLDGLR**AQD DYNGWDINTP

101 AFEEYYQSGL SVIMPVGGQS SFYTDWYQPS QSNGQNYTYK **WETFLTREMP**

151 **AWLQANK**GVS PTGNAAVGLS MSGGSALILA AYYPQQFPYA ASLSGFLNPS

201 EGWWPTLIGL AMNDSGGYNA NSMWGPSSDP AWK**RNDPMVQ IPR**LVANNTR

251 **IWVYCGNGTP SDLGGDNIPA KFLEGLTLRT NQTFRDTYAA DGGR**NGVFNF

301 PPNGTHSWPY WNEQLVAMK**A DIQHVLNGAT PPAAPAAPAA**

③ Thioredoxin

Protein sequence coverage: 36%

Matched peptides shown in ***bold red***.

1 MTDSEKSATI KVTDASFATD VLSSNKPVLV DFWATWCGPC K**MVAPVLEEI**

51 **ATERATDLTV AKLDVDTNPE TAR**NFQVVSI PTLILFKDGQ PVKRIVGAKG

101 KAALLR**ELSD VVPNLN**

④ PstS1

Protein sequence coverage: 53%

Matched peptides shown in ***bold red***.

1 MKIRLHTLLA VLTAAPLLLA AAGCGSKPPS GSPETGAGAG TVATTPASSP

51 VTLAETGSTL LYPLFNLWGP AFHERYPNVT ITAQGTGSGA GIAQAAAGTV

101 NIGASDAYLS EGDMAAHK**GL MNIALAISAQ QVNYNLPGVS EHLK**LNGK**VL**

151 **AAMYQGTIKT WDDPQIAALN PGVNLPGTAV VPLHRSDGSG DTFLFTQYLS**

201 **KQDPEGWGK**S PGFGTTVDFP AVPGALGENG NGGMVTGCAE TPGCVAYIGI

251 SFLDQASQR**G LGEAQLGNSS GNFLLPDAQS IQAAAAGFAS KTPANQAISM**

301 **IDGPAPDGYP IINYEYAIVN NR**QK**DAATAQ TLQAFLHWAI TDGNKASFLD**

351 **QVHFQPLPPA VVKLSDALIA TISS**

⑤ Rv3803c

Protein sequence coverage: 46%

Matched peptides shown in ***bold red***.

1 MKGRSALLRA LWIAALSFGL GGVAVAAEPT AK**AAPYENLM VPSPSMGR**DI

51 PVAFLAGGPH AVYLLDAFNA GPDVSNWVTA GNAMNTLAGK **GISVVAPAGG**

101 **AYSMYTNWEQ DGSKQWDTFL SAELPDWLAA NRGLAPGGHA AVGAAQGGYG**

151 **AMALAAFHPD R**FGFAGSMSG FLYPSNTTTN GAIAAGMQQF GGVDTNGMWG

201 APQLGRWK**WH DPWVHASLLA QNNTRVWVWS PTNPGASDPA AMIGQAAEAM**

251 **GNSRMFYNQY R**SVGGHNGHF DFPASGDNGW GSWAPQLGAM SGDIVGAIR

⑥ Rv2878c

Protein sequence coverage: 38%

Matched peptides shown in ***bold red***.

1 MSLRLVSPIK AFADGIVAVA IAVVLMFGLA NTPRAVAADE RLQFTATTLS

51 GAPFDGASLQ GKPAVLWFWT PWCPFCNAEA PSLSQVAAAN PAVTFVGIAT

101 **RADVGAMQSF VSKYNLNFTN LNDADGVIWA RYNVPWQPAF VFYRADGTST**

151 **FVNNPTAAMS QDELSGR**VAA LTS

⑦ Ag85B

Protein sequence coverage: 26%

Matched peptides shown in ***bold red***.

1 MTDVSRKIRA WGRRLMIGTA AAVVLPGLVG LAGGAATAGA FSRPGLPVEY

51 LQVPSPSMGR DIK**VQFQSGG NNSPAVYLLD GLR**AQDDYNG WDINTPAFEW

101 YYQSGLSIVM PVGGQSSFYS DWYSPACGK**A GCQTYKWETF LTSELPQWLS**

151 **ANR**AVKPTGS AAIGLSMAGS SAMILAAYHP QQFIYAGSLS ALLDPSQGMG

201 PSLIGLAMGD AGGYK**AADMW GPSSDPAWER NDPTQQIPKL VANNTR**LWVY

251 CGNGTPNELG GANIPAEFLE NFVRSSNLKF QDAYNAAGGH NAVFNFPPNG

301 THSWEYWGAQ LNAMK**GDLQS SLGAG**

⑧ Rv1926c

Protein sequence coverage: 57%

Matched peptides shown in ***bold red***.

1 MKLTTMIKTA VAVVAMAAIA TFAAPVALAA YPITGK**LGSE LTMTDTVGQV**

51 **VLGWK**VSDLK **SSTAVIPGYP VAGQVWEATA TVNAIRGSVT PAVSQFNART**

101 **ADGINYRVLW QAAGPDTISG ATIPQGEQST GK**IYFDVTGP SPTIVAMNNG

151 MEDLLIWEP

SDS-PAGE analysis of native purified *M. tuberculosis* proteins and their mass spectrometry analyses are shown.

M, protein molecular standards, ① Rv1860; ② Ag85C; ③ Thioredoxin; ④ PstS1; ⑤ Rv3803c; ⑥ Rv2878c; ⑦ Ag85B; ⑧ Rv1926c. Identified amino acid sequence are marked in red

Supplementary Fig. S3

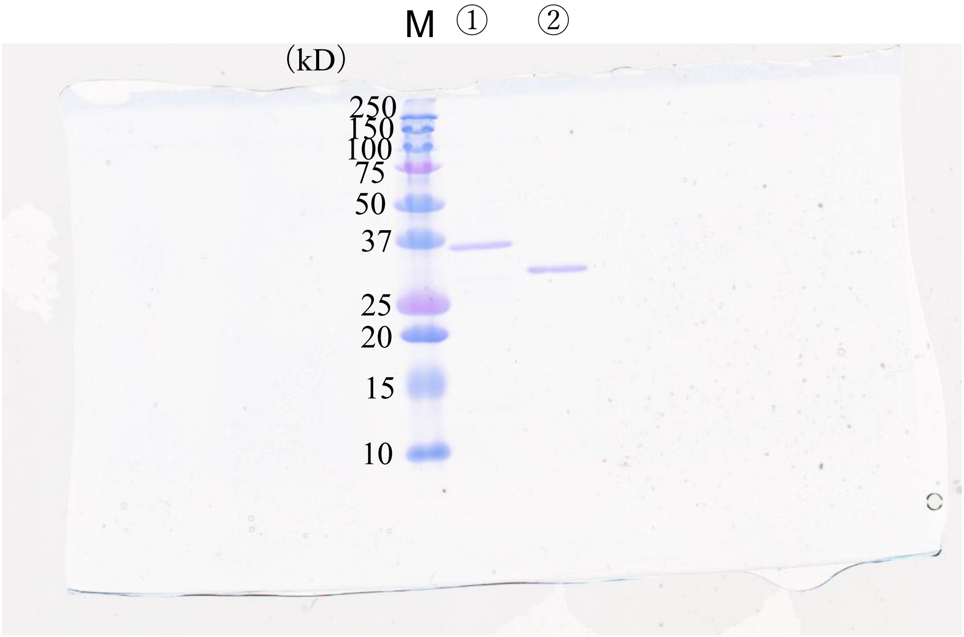

① ePstS1

Protein sequence coverage: 66%

Matched peptides shown in ***bold red***.

```
  1 MKIRLHTLLA VLTAAPLLLA AAGCGSKPPS GSPETGAGAG TVATTPASSP
 51 VTLAETGSTL LYPLFNLWGP AFHERYPNVT ITAQGTGSGA GIAQAAAGTV
101 NIGASDAYLS EGDMAAHKGL MNIALAISAQ QVNYNLPGVS EHLKLNGKVL
151 AAMYQGTIKT WDDPQIAALN PGVNLPGTAV VPLHRSDGSG DTFLFTQYLS
201 KQDPEGWGKS PGFGTTVDFP AVPGALGENG NGGMVTGCAE TPGCVAYIGI
251 SFLDQASQRG LGEAQLGNSS GNFLLPDAQS IQAAAAGFAS KTPANQAISM
301 IDGPAPDGYP IINYEYAIVN NRQKDAATAQ TLQAFLHWAI TDGNKASFLD
351 QVHFQPLPPA VVKLSDALIA TISS
```

② eAg85B

Protein sequence coverage: 46%

Matched peptides shown in ***bold red***.

```
  1 MTDVSRKIRA WGRRLMIGTA AAVVLPGLVG LAGGAATAGA FSRPGLPVEY
 51 LQVPSPSMGR DIKVQFQSGG NNSPAVYLLD GLRAQDDYNG WDINTPAFEW
101 YYQSGLSIVM PVGGQSSFYS DWYSPACGKA GCQTYKWETF LTSELPQWLS
151 ANRAVKPTGS AAIGLSMAGS SAMILAAYHP QQFIYAGSLS ALLDPSQGMG
201 PSLIGLAMGD AGGYKAADMW GPSSDPAWER NDPTQQIPKL VANNTRLWVY
251 CGNGTPNELG GANIPAEFLE NFVRSSNLKF QDAYNAAGGH NAVFNFPPNG
301 THSWEYWGAQ LNAMKGDLQS SLGAG
```

SDS-PAGE analysis of recombinant *M. tuberculosis* proteins from *E. coli* and the mass spectrometry analyses of ePstS1 and eAg85B are shown.
M, protein molecular standards, ① ePstS1; ② eAg85B. Identified amino acid sequence are marked in red.

Supplementary Fig. S4

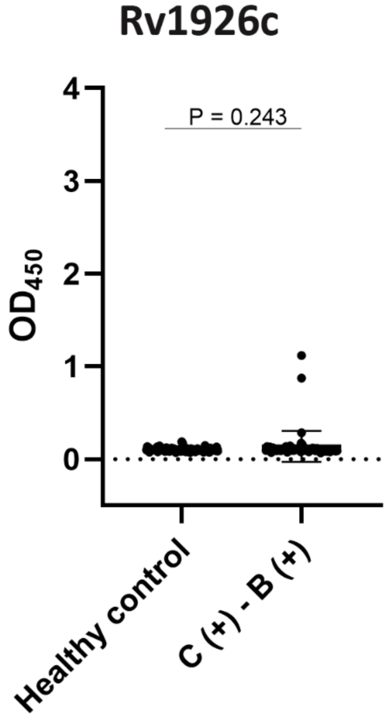

Graphic of the IgGs amount. ELISA optical density of antigen Rv1926c indicating the serum antibody concentration in the clinically positive – bacteriology test positive [C (+) - B (+)] and HC groups. The results were analyzed as individually and the data presented as mean ± SD
